# Impact of concomitant DMARD therapy on adherence to treatment with etanercept and infliximab in rheumatoid arthritis. Results from a six-year observational study in southern Sweden

**DOI:** 10.1186/ar2084

**Published:** 2006-11-22

**Authors:** Lars Erik Kristensen, Tore Saxne, Jan-Åke Nilsson, Pierre Geborek

**Affiliations:** 1 Department of Rheumatology, Lund University Hospital, Kioskgatan 3, SE-221 85 Lund, Sweden

## Abstract

The objective of this work is to compare the adherence to therapy of patients receiving etanercept and infliximab during first tumour necrosis factor (TNF)-blocking treatment course in rheumatoid arthritis. Special emphasis is placed on potential predictors for treatment termination and the impact of concomitant methotrexate (MTX) or other disease-modifying antirheumatic drugs (DMARDs). Patients (*n *= 1,161) with active rheumatoid arthritis, not responding to at least two DMARDs including MTX starting etanercept or infliximab therapy for the first time, were included in a structured clinical follow-up protocol. Information on diagnosis, disease duration, previous and ongoing DMARDs, treatment start and termination, as well as cause of withdrawal was prospectively collected during the period of March 1999 through December 2004. Patients were divided into six groups according to TNF-blocking drugs and concomitant DMARDs. Five-year level (one-year) of adherence to therapy was 36% (69%) for patients receiving infliximab in combination with MTX compared with 65% (89%) for patients treated with etanercept and MTX (*p *< 0.001). Cox regression models showed that the risk for premature treatment termination of patients treated with infliximab was threefold higher than for etanercept (*p *< 0.001). Also, the regression analysis showed that patients receiving concomitant MTX had better treatment continuation than patients treated solely with TNF blockers (*p *< 0.001). Moreover, patients receiving concomitant MTX had superior drug survival than patients receiving other concomitant DMARDs (*p *< 0.010). The superior effect of MTX was associated primarily with fewer treatment terminations because of adverse events. In addition, the study identifies low C-reactive protein level, high age, elevated health assessment questionnaire score, and higher previous number of DMARDs as predictors of premature treatment termination. In summary, treatment with etanercept has higher adherence to therapy than treatment with infliximab. Concomitant MTX is associated with improved treatment continuation of biologics when compared with both TNF blockers as monotherapy and TNF blockers combined with other DMARDs.

## Introduction

During the past decade, the armamentarium of effective antirheumatic drugs has increased considerably. Nevertheless, a definite remission-inducing drug has yet to be discovered, and the vast majority of rheumatoid arthritis (RA) patients are dependent on lifelong treatment in order to suppress joint damage and functional impairment. The efficacy and tolerability of tumour necrosis factor (TNF) blockers have so far been studied in randomised controlled clinical trials (RCTs) and observational studies [1-10]. However, data directly comparing different anti-TNF treatments and disease-modifying antirheumatic drug (DMARD) combinations are still sparse.

This paper reports six-year data from an observational study using a structured clinical protocol developed by the South Swedish Arthritis Treatment Group (SSATG) [11]. The objective is to investigate predictors associated with premature TNF-blocker treatment termination. Thus, we compare the adherence to therapy of etanercept and infliximab in biologic-naïve RA patients treated in clinical practice. Emphasis is placed on the impact of concomitant methotrexate (MTX) or other DMARDs and patient characteristics at treatment initiation.

## Materials and methods

### Patients

**Figure 1:**
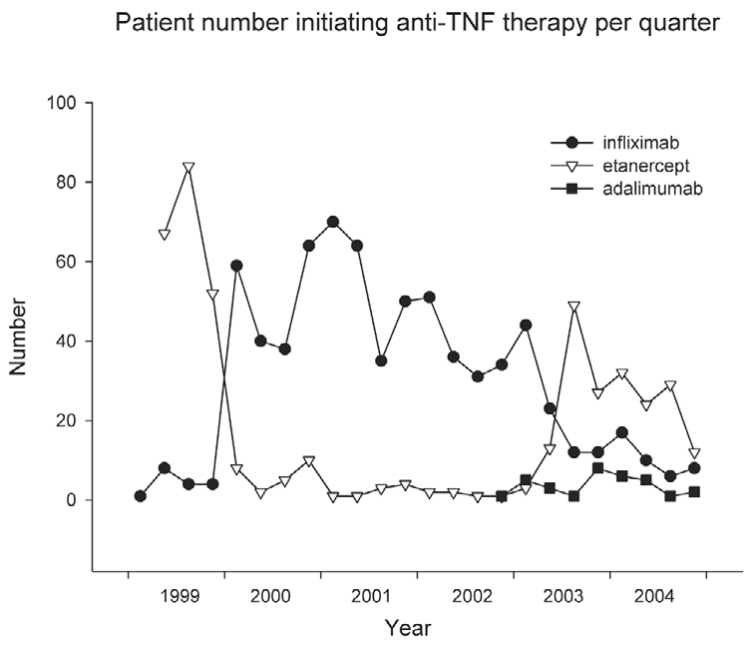
Number of patients starting anti-tumour necrosis factor (TNF) therapy during the observational period. The figure presents the number of rheumatoid arthritis patients, per quarter, starting infliximab, etanercept, or adalimumab for the first time during the period of 1999 through 2004 in southern Sweden.

Data were collected in a database following a structured clinical protocol designed for drug monitoring. As the protocol was designed to meet the legislative documentation required in Sweden, no formal approval from the ethical committee was necessary. Patients eligible for the study had a diagnosis of RA according to clinical judgement by the treating physician. A review showed that 98% of the patients fulfilled the American College of Rheumatology 1987 classification criteria for RA. The patients were treated at eight hospital centers in southern Sweden serving a population of about 1.3 million during the period of March 1999 through December 2004. During the entire study period, patients were continually enrolled. A review of anti-TNF drugs sold in pharmacies compared with patients registered in our database revealed that approximately 90% of the patients receiving these drugs in southern Sweden were included in the database [12]. Subjects eligible for TNF-blocking therapy were selected by physicians based on disease activity and/or unacceptable steroid use. No formal level of disease activity was required; however, the patients should have received at least two DMARDs, including MTX previously without satisfactory response. Selection of a particular drug was based primarily on availability. The access to infliximab was limited in 1999, whereas supply of etanercept was limited during the period of February 2000 through June 2003 (Figure 1). Patients having received biologic therapy prior to inclusion were excluded from this study. Thus, only patients receiving their first treatment course of biologic therapy (biologic-naïve patients) were enrolled in the analysis.

Treatment guidelines encouraged infliximab to be administered in combination with MTX. However, when infliximab was introduced in Sweden, it was not obvious that co-medication with MTX was needed and that is why a substantial number of our infliximab patients were treated without concomitant MTX. Dosages of the TNF-blocking agents followed the recommendations of the manufacturers. Etanercept 25 mg subcutaneously was administered twice a week. Infliximab was infused at 3 mg/kg at 0, 2, and 6 weeks and then every eighth week. Depending on primary or secondary failure, the dosage of infliximab could be increased in increments of 100 mg to a maximum of 500 mg administered at 4- to 8-week intervals.

### Method

Clinical data were prospectively collected at 0, 3, 6, and 12 months and subsequently every 3 to 6 months. No patients were excluded for lack of registrations at any follow-up times. At inclusion, the following data were recorded: year of disease onset, previous and concomitant DMARD treatment, and systemic prednisolone dosage. At inclusion and at each follow-up visit, the following clinical data were registered: health assessment questionnaire (HAQ) score, patient-scored visual analogue scale for pain (VASpain) and general health (VASglobal), physician's global assessment of disease activity on a five-grade scale (EVALglobal), 28 tender and swollen joint count, erythrocyte sedimentation rate, and C-reactive protein (CRP). The 28-joint disease activity score (DAS28) was also calculated [13].

Any withdrawal from treatment was registered prospectively and classified by the treating physician as withdrawal caused by adverse events (AEs), lack of response/treatment failure, or miscellaneous. No formal criteria were required to terminate treatment, and the decision was based on the judgement of the treating physician. The category 'miscellaneous' consisted mainly of patients with poor compliance or subjects moving away from southern Sweden. In approximately 2% of the withdrawal cases, the cause of cessation was registered as both treatment failure and AE; for these patients, the cause of withdrawal was subsequently classified as an AE. Patients stopping treatment prior to surgery were considered to remain on therapy if the duration of the pause did not exceed 12 months.

For study of the impact of concomitant DMARDs, the patients were divided into six groups. Two main groups of patients received etanercept or infliximab. These groups were further subdivided into three groups depending on concomitant DMARD use at TNF-blocking treatment initiation: one group of patients receiving MTX, another group of patients receiving other DMARDs, and finally a group of patients treated with TNF blockers as the only antirheumatic therapy (monotherapy). The group 'other DMARD' consisted of patients receiving hydroxychloroquine, sulfasalazine, and (to a lesser extent) leflunomide and azathioprine.

In an open study design, there is a possibility for selection bias favouring one drug over another. Therefore, a subgroup analysis was performed including patients from 1999 through June 2001 with a follow-up time of 2 years (until June 2003). Because of limited access of TNF-blocking drugs during this period (Figure 1), analyses of treatments were possible without selection bias as well as during a 2-year observational period with restricted access of TNF-blocking agents. After June 2003, infliximab, etanercept, as well as adalimumab were widely available (Figure 1). The number of biologic-naïve patients starting adalimumab was too limited to allow meaningful analysis, and patients treated with adalimumab were therefore excluded from this report.

Furthermore, a linkage between the SSATG database and the Swedish in-hospital discharge register was performed to identify possible differences in co-morbidities at treatment initiation for the infliximab and the etanercept groups. Patients initiating therapy were checked for an up-to-10-year prior history of cardiovascular diseases, any malignancies, chronic pulmonary diseases, and diabetes according to ICD (International Statistical Classification of Diseases and Related Health Problems) 9 or 10 diagnosis codes. Ethical approval was obtained before making the register linkage.

To minimise follow-up loss, data for all patients included were checked every 6 months. In case of incomplete clinical data for a period of more than 250 days, a request to report the missing data or provide the time and reason for potential drug discontinuation was sent to the treating physician. Less than 2% of the patients remained with unknown treatment status when we retrieved results for this study.

### Statistical analysis

Baseline clinical characteristics were analysed by the Kruskal-Wallis and Mann-Whitney *U *tests for comparison of groups for continuous variables, whereas the χ^2 ^test was used for categorical variables. Values are reported as the mean ± standard deviation except where stated otherwise. Adherence to therapy data was estimated according to Kaplan-Meier and further analysed with log-rank statistics for comparing different treatments. Cox proportional hazard models were used to investigate the effect of possible risk factors for treatment termination (age, gender, disease duration, HAQ score, DAS28, CRP level, number of previous DMARDs, year of treatment initiation, and concomitant DMARDs). These models were based on all patients included in the study except where stated otherwise. Moreover, time-dependent Cox proportional hazard models were performed using covariates that might change over time (HAQ score, CRP level, and DAS28) to complement findings from the predictor analysis. The model assumptions for using these regression analyses were tested and found valid. Level of significance was chosen to be *p *< 0.05.

## Results

### Baseline data

During the observational period, a total of 1,161 patients were enrolled in the study. Demographic data and clinical characteristics of patients studied are summarised in Table 1. Notably, only two significant differences were found when comparing the respective subgroups of infliximab and etanercept. Patients receiving infliximab as monotherapy were older than patients receiving etanercept as monotherapy. Patients treated with concomitant MTX received a significantly lower weekly dose when treated with infliximab compared with etanercept (14.3 mg/week versus 16.1 mg/week).

When comparing baseline data for the different subgroups (concomitant MTX, other DMARD combination, or TNF-blocking agent as monotherapy), several significant differences were found. In general, patients receiving concomitant MTX had lower HAQ, VASglobal, VASpain, and DAS28 scores when compared with other subgroups. Patients in the MTX groups were overall younger, had shorter disease duration, and fewer previous DMARDs. Finally, patients receiving other DMARDs or TNF blockers as monotherapy had a trend for higher CRP levels. Thus, the main differences in baseline characteristics were found between subgroups of patients and not between patients receiving infliximab or etanercept.

The register linkage did not show any significant differences in discharge diagnosis for patients initiated on infliximab compared with etanercept. Thus, patients eligible for therapy had a 10-year prior hospitalisation history of cardiovascular disease in 7% versus 6%, diabetes in 5% versus 5%, any malignancies in 4% versus 3%, and chronic pulmonary disease in 4% versus 4% for infliximab- and etanercept-treated patients, respectively.

Levels of adherence to therapy for infliximab and etanercept with concomitant MTX at 5-year (4-year; 1-year) follow-up were 36% (38%; 69%) and 65% (75%; 89%), respectively. When infliximab and etanercept were administered as monotherapy, the levels of adherence to therapy at 4 years (1 year) were 18% (47%) and 53% (74%), respectively. The corresponding numbers for TNF blockers given in combination with other DMARDs were 27% (58%) and 71% (85%) for infliximab and etanercept, respectively.

**Figure 2:**
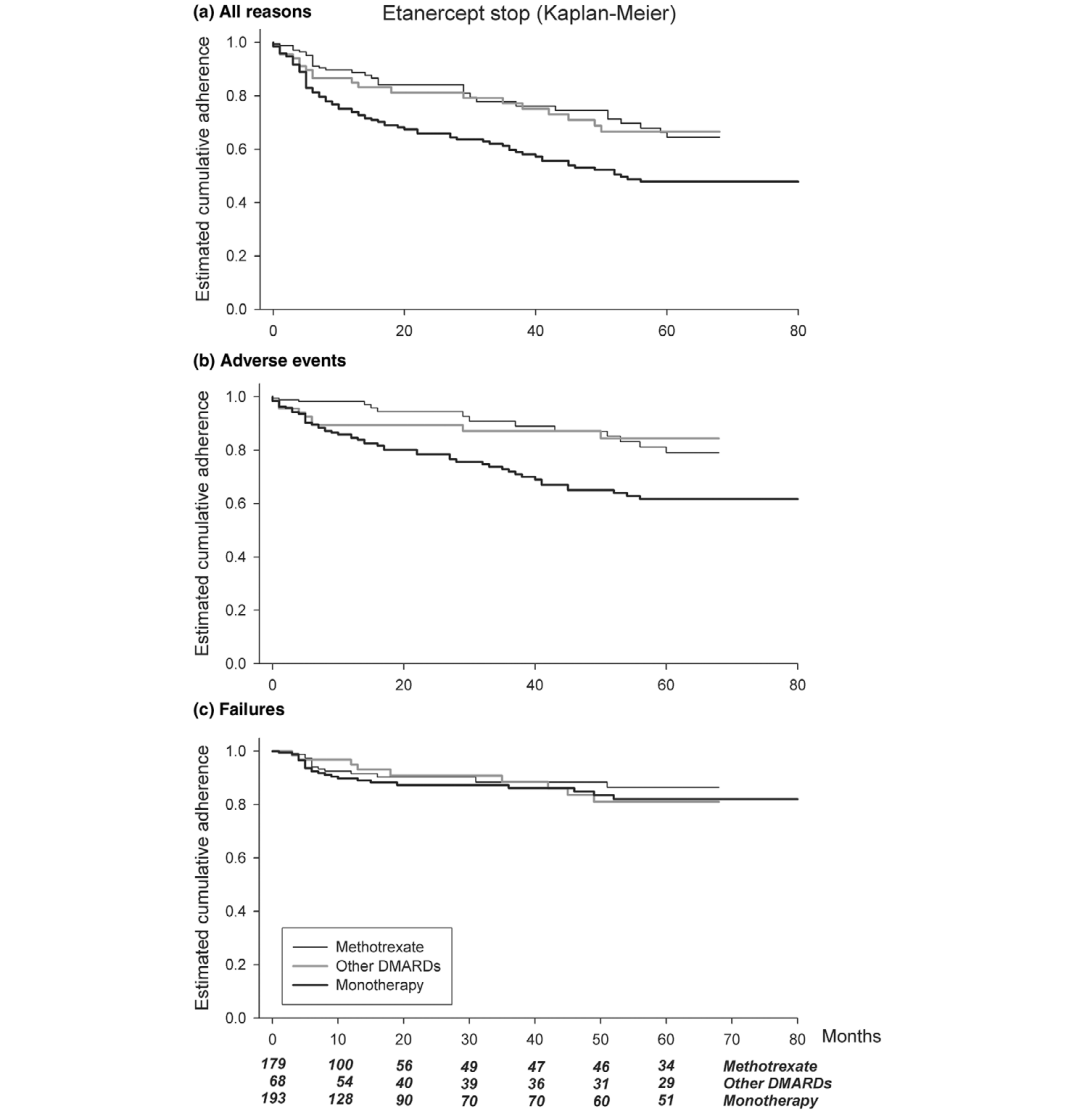
Adherence to therapy for patients treated with etanercept. The level of adherence to therapy is shown as the fraction (between 1 and 0) of patients remaining on therapy during the observation period. Withdrawal due to any reason **(a)**, adverse events **(b)**, or failure to treatment **(c) **is presented separately. The number of patients under observation at each time point is listed at the bottom of the figure. DMARD, disease-modifying antirheumatic drug.

**Figure 3:**
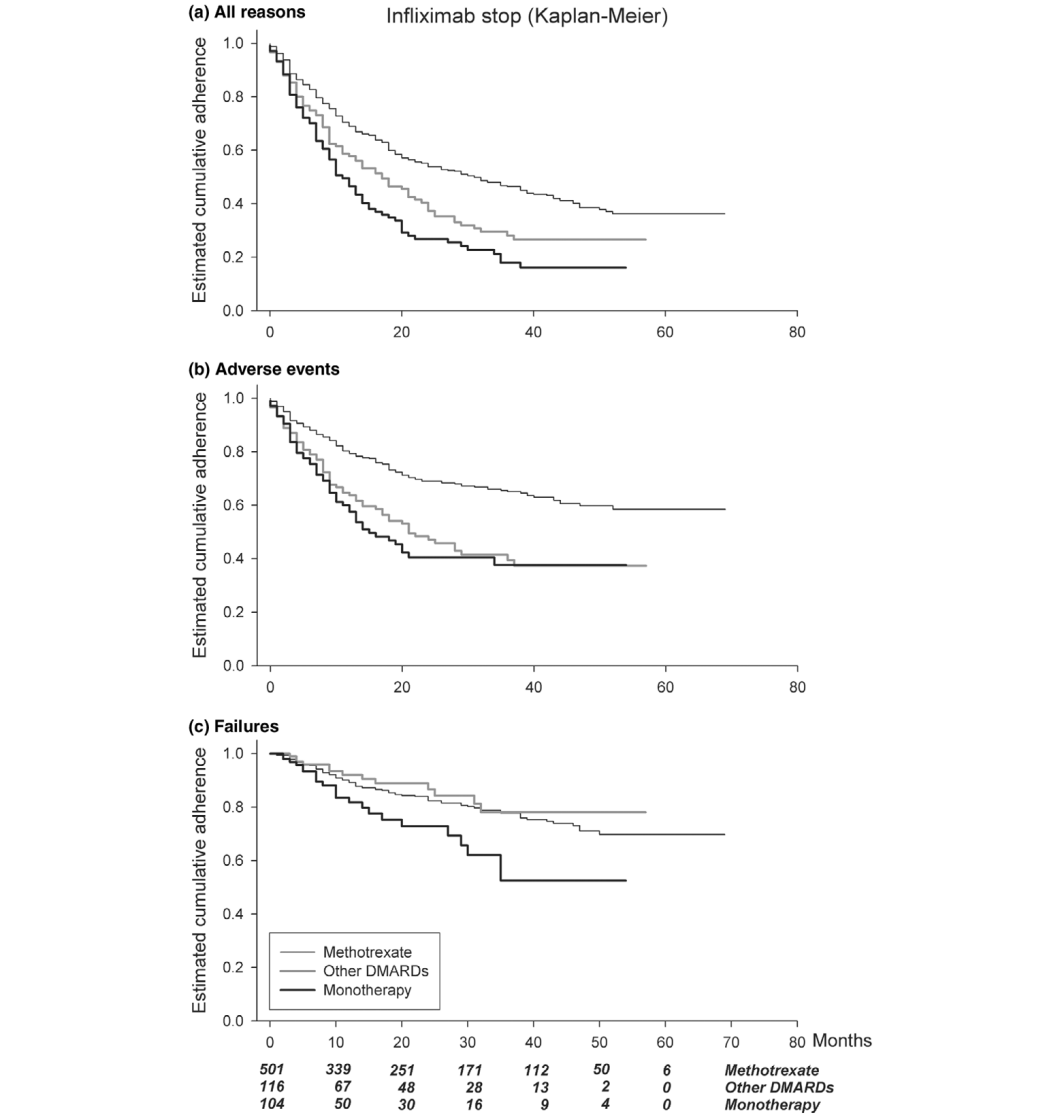
Adherence to therapy for patients treated with infliximab. The level of adherence to therapy is shown as the fraction (between 1 and 0) of patients remaining on therapy during the observation period. Withdrawal due to any reason **(a)**, adverse events **(b)**, or failure to treatment **(c) **is presented separately. The number of patients under observation at each time point is listed at the bottom of the figure. DMARD, disease-modifying antirheumatic drug.

Levels of total adherence to therapy for all subgroups are shown in Figures 2 to 3a, whereas survival curves for withdrawal from treatment due to AEs and treatment failure are shown in Figures 2 to 3b and 2 to 3c, respectively. Infliximab showed significantly lower total adherence to therapy compared with etanercept for all subgroups (*p *< 0.001).

Figures 2 to 3b show that infliximab has more withdrawals because of AEs when compared with etanercept in all three subgroups (*p *< 0.001). However, when studying withdrawals due to treatment failure (Figures 2 to 3c), the differences between infliximab and etanercept were less obvious and only significant for the subgroups of patients receiving MTX (*p *= 0.026) and monotherapy (*p *= 0.002). Thus, AEs are mainly what account for the differences seen in total adherence to therapy between etanercept and infliximab.

Patients receiving concomitant MTX showed significantly higher levels of total adherence to therapy when compared with monotherapy both in the infliximab and the etanercept groups (*p *< 0.001). Also, patients treated with infliximab experienced significantly better adherence to therapy when treated with MTX (*p *= 0.002) compared with other concomitant DMARDs. No such difference was found for etanercept. In the etanercept group, total adherence to therapy was significantly higher for the subgroup receiving concomitant DMARDs compared with monotherapy (*p *= 0.015).

Patients receiving MTX had fewer dropouts due to AEs when compared with patients receiving biologic therapy as monotherapy (*p *< 0.001). Also, patients receiving infliximab with concomitant MTX showed fewer withdrawals because of AEs when compared with patients receiving other concomitant DMARDs (*p *< 0.001). No such difference was found for patients receiving etanercept. When the impact of DMARDs on withdrawal due to treatment failure was studied, patients in the infliximab group receiving monotherapy had higher withdrawal compared with patients receiving MTX (*p *= 0.019) or other concomitant DMARDs (*p *= 0.011). Patients receiving etanercept did not show these differences.

There were no differences at treatment termination in disease activity, measured by DAS28, between patients terminating treatment because of failure in the infliximab groups (DAS28 = 5.1 [95% confidence interval (CI) 4.9; 5.4]) compared with the etanercept groups (DAS28 = 5.1 [95% CI 4.8; 5.4]).

Patients withdrawing from treatment because of miscellaneous reasons comprised only a small percentage of all withdrawals, and no statistical dissimilarities were found between the different treatment groups (data not shown).

### Regression analysis

Significant differences in baseline characteristics between subgroups (Table 1) made it necessary to perform a Cox proportional hazard regression analysis to elucidate whether these differences influenced adherence to therapy. Table 2 shows hazard ratios (HRs) and 95% CIs for the regression analysis. When differences in baseline data were adjusted for, MTX was shown to protect against premature treatment termination when compared with other concomitant DMARDs and monotherapy. Also, all patients treated with infliximab had a threefold increased risk of stopping therapy when compared with etanercept. Furthermore, using HAQ score, CRP level, and DAS28 as time-dependent covariates showed similar findings with only minor decrements in differences described above (Table 2). The reason for better overall adherence to therapy with concomitant MTX appeared to be significantly fewer dropouts because of AEs (*p *< 0.001). On the other hand, MTX did not show significant differences compared with other DMARDs and monotherapy when examining dropouts because of treatment failure. No significant statistical interaction was found between subgroup treatment and TNF-blocking agents with regard to level of total adherence to therapy (that is, the differences found between drug survival of infliximab compared with etanercept did not depend on concomitant DMARD treatment).

Subgroup analysis of etanercept and infliximab comparing groups of patients starting either TNF-blocking drugs in combination with MTX or as monotherapy is shown in Table 3. The number of patients initiated on TNF-blocking agents in combination with other DMARDs was too small to allow meaningful subgroup analysis. No significant baseline differences were found when comparing the respective subgroups of infliximab and etanercept for age, gender, DAS28, CRP level, disease duration, and HAQ score (data not shown). The two inclusion periods displayed in Table 3 demonstrate that either infliximab or etanercept was limited in access, thus allowing for treatment initiation of either drug without selection bias. The 1-year level of adherence to therapy in this subgroup was similar to the complete adherence analysis, and when adjusting for age, gender, DAS28, HAQ score, disease duration, previous DMARDs, and CRP level, patients treated with infliximab had a threefold to fourfold increased risk for stopping therapy compared with etanercept (*p *< 0.001). The HRs diminished slightly to 2.75 and 3.15 (*p *< 0.001) when using HAQ score, CRP level, and DAS28 as time-dependent covariates (Table 3). These relative risks were consistent with the results reported for the entire group of patients. The Cox proportional hazard models used in the subgroup analysis were based only on patients included during the period of interest.

### Predictors for drug discontinuation

Independent of anti-TNF treatment, significant predictors of premature treatment termination were higher age (HR = 1.11; CI = 1.01; 1.22), higher HAQ score (HR = 1.17; CI = 1.06; 1.30), and higher previous number of DMARDs at inclusion (HR = 1.13; CI = 1.02; 1.25). In contrast, high CRP level at treatment initiation was a predictor for prolonged drug adherence (HR = 0.90; CI = 0.81; 0.98). Gender, year of treatment initiation, DAS28, and disease duration prior to treatment initiation did not predict the level of adherence to therapy.

## Discussion

The results presented in this study demonstrate a higher level of adherence to therapy for etanercept compared with infliximab. This difference was significant for patients treated with concomitant MTX, other concomitant DMARDs, as well as for patients treated with TNF-blocking drugs only. Adjusting for potential confounding factors further accentuated the difference. This result is in agreement with data recently presented by Gomez-Reino and coworkers [14]. However, information on concomitant DMARDs, HAQ scores, and disease activity was not available in the study by Gomez-Reino and coworkers, making direct comparison of absolute HRs and levels of adherence to therapy difficult.

The levels of adherence to therapy found in this study are somewhat higher than data recently presented by Zink and coworkers [10]. One explanation for this could be that our material includes only patients receiving their first treatment course of biologic therapy whereas the adherence data presented by Zink and coworkers also includes patients previously treated with other biological therapy. In addition, CRP levels in patients enrolled in our study were considerably higher than in patients studied by Zink and coworkers (30 to 41 mg/l compared with 20 mg/l), and as shown in this paper, high CRP levels might be a predictor for a high level of adherence to therapy. On the other hand, the 1-year level of adherence to therapy found in our study closely resembles values found in randomised controlled clinical trials. We found 1-year level of adherence to therapy for patients treated with infliximab and concomitant MTX to be approximately 70% compared with 73% in the ATTRACT (Anti-TNF Trial in Rheumatoid Arthritis with Concomitant Therapy) trial [2]. Also, patients treated with etanercept showed a 74% 1-year level of adherence to therapy compared with 76% in the TEMPO (Trial of Etanercept and Methotrexate with Radiographic Patient Outcomes) trial when given as monotherapy [9]. When etanercept was given in combination with MTX, the corresponding numbers were 89% compared with 84%. However, great methodological differences exist between RCTs and observational studies, making direct comparisons difficult to interpret [15].

In accordance with previous studies, we found that patients treated with concomitant MTX experienced a higher adherence to therapy [8,10] compared with patients treated with anti-TNF agents only. Moreover, our data suggest that MTX is superior to concomitant DMARDs such as hydroxychloroquine, sulfasalazine, and leflunomide in preventing treatment discontinuation of TNF blockers. This difference was less clear for patients treated with etanercept when examining unadjusted adherence data in Figure 2a. However, when adjusting for heterogeneity at baseline, a difference was found (Table 2). A possible explanation for the apparent superiority of concomitant MTX may be that MTX is a more potent antirheumatic drug in itself. Another reason could be that patients not tolerating MTX also possess yet-undefined characteristics or co-morbidities predisposing to lower levels of adherence to anti-TNF therapy. Nevertheless, our data suggest that patients who tolerate MTX well also are likely to tolerate TNF-blocking agents. Thus, tolerance to MTX therapy may be used as a surrogate measure for potential high tolerability of anti-TNF treatments in clinical practice.

Our study identifies withdrawal due to AEs as the main cause of differences in total adherence to therapy between infliximab and etanercept. In addition, the positive impact of concomitant MTX was also associated primarily with fewer dropouts because of AEs. One mechanism underlying this difference could be the formation of neutralising and immunopathogenic antibodies against the murine part of infliximab, leading to a higher number of AEs [16]. It can also be speculated that MTX in turn can effectively inhibit the formation of these immunopathogenic antibodies, thus decreasing the risk for AEs [16-18]. Moreover, various data regarding differences in mechanism of action between infliximab and etanercept are emerging [19-21]. These findings could possibly explain differences demonstrated in this study.

At this point, it should be noted that reasons for ceasing treatment are scored by the treating physician, and inter-observer variation in the classification of the stop reason therefore exists. The reason for the low percentages of termination because of both treatment failure and AEs is due primarily to the design of the registration scheme and should not be interpreted as certainty of the termination reason. Thus, results regarding cause of treatment termination must be interpreted with care, placing more emphasis on overall adherence to therapy [10]. Although potential bias also exists when using this measure in drug studies [22], the wide use of this pragmatic measure makes it suitable for this report and further inter-trial comparisons.

We identify high age, elevated HAQ score, and higher previous number of DMARDs as significant predictors of premature treatment termination. In contrast, high CRP level at inclusion seemed to protect against treatment termination.

Patients with a high level of systemic inflammation may have a larger potential for improvement in clinical status when an immunosuppressive drug such as a TNF-blocking agent is administered. This may explain the better drug adherence observed in patients with high CRP level at inclusion.

The open non-randomised nature of this study may induce bias in the process of selecting patients for particular treatments and the subsequent collection of data [23-25]. All data entries were centralised to ensure uniform interpretation of registration forms. Confounding by indication cannot be excluded from this study. Consequently, a Cox regression model was applied to adjust for differences in baseline. This adjustment increased the HR for terminating treatment of infliximab compared with etanercept from 2.37 to 2.92 (Table 2), indicating that potential selection bias might have favoured prescription of infliximab over etanercept. Consistently, the findings were supported by time-dependent Cox proportional hazard models using covariates that might change over time (HAQ score, CRP level, and DAS28). Moreover, a subgroup analysis was performed comparing unselected treatment initiation of etanercept and infliximab (Table 3). The result of this analysis was consistent with the overall survival analysis both in relation to absolute adherence values and HRs. Thus, the subgroup analysis showed that the risk for terminating treatment with infliximab was threefold to fourfold higher than for etanercept. It is therefore unlikely that selection bias has had substantial impact on the results presented in this study. Moreover, only minor differences were found between the main therapies at baseline, making selection bias less likely. Also, linkage to in-hospital discharge register showed no baseline difference in prior diabetes, malignancies, chronic pulmonary diseases, and cardiovascular diseases when comparing patients treated with etanercept with those receiving infliximab. Patients entering the subgroup analysis were followed only until June 2003. During this period, the availability of TNF-blocking drugs was limited (Figure 1), and switching of therapy because of convenience was therefore minimised.

The National Healthcare System in Sweden provides free access to TNF-blocking drugs if prescribed by the treating physician, regardless of the social status of the patient. Thus, potential differences in costs of the TNF-blocking agents did not induce a selection bias.

Also, the possibility for termination bias was investigated by examining disease activity of patients terminating therapy because of treatment failure. We did not find any differences in the level of disease activity when comparing infliximab with etanercept, and bias in the withdrawal of patients because of treatment failure is therefore unlikely. Finally, at present there are only sparse data directly comparing the different biologic drugs [25], thus giving no obvious reason for favouring prescription of one drug over another.

## Conclusion

This study indicates that treatment with etanercept leads to higher adherence to therapy than treatment with infliximab. Concomitant MTX is associated with improved treatment continuation of biologics when compared with both TNF blockers as monotherapy and TNF blockers combined with other DMARDs. Thus, our data suggest that patients eligible for TNF-blocking treatment have a better chance of long adherence to therapy if they are tolerating MTX and started on etanercept rather than infliximab. Treating patients with low CRP levels, high age, elevated HAQ score, and high previous number of DMARDs may lead to premature treatment termination.

## Abbreviations

AE = adverse event; CI = confidence interval; CRP = C-reactive protein; DAS28 = 28-joint disease activity score; DMARD = disease-modifying antirheumatic drug; HAQ = health assessment questionnaire; HR = hazard ratio; MTX = methotrexate; RA = rheumatoid arthritis; RCT = randomised controlled trial; SSATG = South Swedish Arthritis Treatment Group; TNF = tumour necrosis factor; VASglobal = visual analogue scale for general health; VASpain = visual analogue scale for pain.

## Competing interests

LEK has received a fee for speaking by Wyeth (Madison, NJ, USA), the manufacturer of etanercept. PG has received fees for speaking by Abbott Laboratories (Abbott Park, IL, USA), Schering-Plough Corporation (Kenilworth, NJ, USA), and Wyeth, the manufacturers of adalimumab, infliximab, and etanercept, respectively. The sum of all fees adds up to less than $10,000 USD per person. Besides the above, all authors have nothing else to declare.

## Authors' contributions

LEK (guarantor)participated in study design, acquisition of data, draft and revision ofthe manuscript, and analysis and interpretation of data. TS participated in study design, revision ofthe manuscript, and interpretation of data. J-AN participated in study design, revision ofthe manuscript, and analysis and interpretation of data. PG participated in study design, acquisition of data, revision ofthe manuscript, and interpretation of data. All authors read and approved the final manuscript.

## Figures and Tables

**Table 1:** Demographic and clinical characteristics at baseline

	Infliximab I			Etanercept II			Level of significance
	MTX A	Other DMARD B	Mono-therapy C	MTX A	Other DMARD B	Mono-therapy C	

Number	501	116	104	179	68	193	
Age (years)	55.0 (13.6)	57.4 (12.0)	61.0 (12.1)	53.4 (12.9)	54.0 (12.4)	57.7 (13.0)	IC vs. IIC, *p *= 0.040 IIA vs. IIC, *p *= 0.003 IA vs. IC, *p *< 0.001 IB vs. IC, *p *= 0.027
Female	74%	75%	78%	78%	84%	84%	
Disease duration (months)	133.5 (113.8)	165.4 (118.5)	192.9 (132.4)	132.8 (107.3)	180.1 (115.1)	185.3 (121.4)	IIA vs. IIC, *p *< 0.001 IIA vs. IIB, *p *< 0.001 IA vs. IB, *p *= 0.002 IA vs. IC, *p *< 0.001
HAQ score	1.34 (0.62)	1.57 (0.58)	1.69 (0.58)	1.30 (0.61)	1.61 (0.58)	1.60 (0.65)	IIA vs. IIB, *p *< 0.001 IIA vs. IIC, *p *< 0.001 IA vs. IB, *p *< 0.001 IA vs. IC, *p *< 0.001
DAS28	5.5 (1.2)	5.8 (1.1)	5.7 (1.2)	5.5 (1.0)	5.8 (1.2)	5.9 (1.1)	IIA vs. IIB, *p *= 0.026 IIA vs. IIC, *p *< 0.001 IA vs. IB, *p *= 0.013
Number of previous DMARDs	3.1 (1.7)	4.2 (2.2)	4.2 (1.9)	3.1 (1.1)	4.2 (1.8)	4.5 (2.1)	IIA vs. IIB, *p *< 0.001 IIA vs. IIC, *p *< 0.001 IA vs. IB, *p *< 0.001 IA vs. IC, *p *< 0.001
VASglobal (mm)	60 (22)	70 (18)	67 (22)	60 (22)	71 (17)	66 (21)	IIA vs. IIB, *p *= 0.001 IIA vs. IIC, *p *= 0.017 IA vs. IB, *p *< 0.001 IA vs. IC, *p *= 0.004
VASpain (mm)	60 (23)	70 (17)	64 (24)	60 (22)	66 (19)	65 (22)	IIA vs. IIC, *p *= 0.020 IA vs. IB, *p *< 0.001
EVALglobal (mm)	57 (23)	57 (22)	58 (23)	56 (24)	56 (21)	60 (24)	IIA vs. IIC, *p *= 0.015
MTX dosage (mg/week)	14.3 (6.0)	-	-	16.1 (5.0)	-	-	IA vs. IIA, *p *< 0.001
CRP (mg/litre)	33.1 (34.0)	39.5 (38.7)	39.1 (36.6)	32.1 (34.5)	38.4 (30.0)	41.6 (38.0)	IIA vs. IIB, *p *= 0.029 IIA vs. IIC, *p *= 0.008
ESR (mm/hour)	35.0 (24.1)	42.1 (26.8)	44.0 (28.2)	34.2 (23.1)	41.9 (25.0)	44.4 (27.1)	

Values are presented as the mean (standard deviation). CRP, C-reactive protein; DAS28, 28-joint disease activity score; DMARD, disease-modifying antirheumatic drug; ESR, erythrocyte sedimentation rate; EVALglobal, physician's global assessment of disease activity on a five-grade scale; HAQ, health assessment questionnaire; MTX, methotrexate; VASglobal, visual analogue scale for general health; VASpain, visual analogue scale for pain.

**Table 2:** Hazard ratios with 95% confidence intervals and levels of significance for stopping treatment

All reasons	HR (95% CI)	Level of significance
Unadjusted infliximab vs. etanercept	2.37 (1.91; 2.93)	*p *< 0.001
^a^Infliximab vs. etanercept	2.92 (2.32; 3.69)	*p *< 0.001
^b^Monotherapy vs. MTX	1.82 (1.45; 2.29)	*p *< 0.001
^b^Other DMARD vs. MTX	1.45 (1.12; 1.87)	*p *< 0.010
^b^Monotherapy vs. other DMARD	1.22 (0.94; 1.61)	*p *= 0.127
All reasons, time-dependent Cox regression analysis	HR (95% CI)	Level of significance
^a^Infliximab vs. etanercept	2.83 (2.27; 3.54)	*p *< 0.001
^b^Monotherapy vs. MTX	1.48 (1.19; 1.85)	*p *< 0.001
^b^Other DMARD vs. MTX	1.33 (1.08; 1.55)	*p *= 0.017
^b^Monotherapy vs. other DMARD	1.10 (0.86; 1.40)	*p *= 0.448
Adverse events	HR (95% CI)	Level of significance
^b^Monotherapy vs. MTX	2.14 (1.61; 2.84)	*p *< 0.001
^b^Other DMARD vs. MTX	1.75 (1.28; 2.04)	*p *= 0.001
^b^Monotherapy vs. other DMARD	1.23 (0.88; 1.71)	*p *= 0.231
Treatment failure	HR (95% CI)	Level of significance
^b^Monotherapy vs. MTX	1.31 (0.86; 1.99)	*p *= 0.215
^b^Other DMARD vs. MTX	1.07 (0.66; 1.73)	*p *= 0.782
^b^Monotherapy vs. other DMARD	1.22 (0.72; 2.06)	*p *= 0.454

^a^Also adjusted for differences in concomitant disease-modifying antirheumatic drugs (DMARDs). ^b^Also adjusted for differences in tumour necrosis factor-blocking treatment. The first row contains unadjusted data, whereas the remaining data were adjusted for differences in age, gender, year of treatment initiation, 28-joint disease activity score (DAS28), health assessment questionnaire (HAQ) score, disease duration, previous DMARDs, and C-reactive protein (CRP) level. Data presented in the second section use HAQ score, DAS28, and CRP level as time-dependent covariates. CI, confidence interval; HR, hazard ratio; MTX, methotrexate.

**Table 3:** Number of patients in subgroup analysis, adherence to therapy, and hazard ratios for stopping treatment

	Patients included February through December 1999	Patients included February 2000 through June 2001	Adherence to therapy after 1 year (2 years)	Hazard ratio (95% CI)	Hazard ratio (95% CI), time-dependent Cox regression analysis
Etanercept and MTX	58	5^a^	90% (87%)	3.27 (1.76; 6.08)	3.15 (1.84; 5.39)
Infliximab and MTX	12^a^	206	68% (60%)		
Etanercept as monotherapy	100	13^a^	77% (69%)	4.26 (2.60; 7.00)	2.75 (1.70; 4.46)
Infliximab as monotherapy	0^a^	56	45% (27%)		

^a^Patients not included in analysis. The hazard ratios were adjusted for differences in age, gender, 28-joint disease activity score (DAS28), health assessment questionnaire (HAQ) score, disease duration, previous disease-modifying antirheumatic drugs, and C-reactive protein (CRP) level. Data presented in the last column use HAQ score, DAS28, and CRP level as time-dependent covariates. For details, see Results. CI, confidence interval; MTX, methotrexate.
